# Squamous cell carcinoma (Marjolin's ulcer) in an orocutaneous fistula of a large mandibular ameloblastoma: a case report

**DOI:** 10.1186/1752-1947-5-396

**Published:** 2011-08-19

**Authors:** Peter M Nthumba

**Affiliations:** 1 Plastic, Reconstructive and Hand Unit, AIC Kijabe Hospital, Kijabe 00220, Kenya

## Abstract

**Introduction:**

Ameloblastomas are rare lesions constituting 1% of all jaw tumors. Oral squamous cell carcinomas are common lesions; these constitute about 90% of all oral cancers. Concurrent tumors consisting of ameloblastoma and squamous cell carcinoma are extremely rare.

**Case presentation:**

This case report describes a 35-year-old African man who presented with a large mandibular tumor with an orocutaneous fistula that was found to be an ameloblastoma on histopathological examination, with concurrent squamous cell carcinoma histology within the fistula. This presentation was consistent with a Marjolin's ulcer within an ameloblastoma.

**Conclusion:**

Ameloblastomas and Marjolin's ulcers require different management strategies. Careful histopathological examination of surgical specimens is key to patient outcome, as treatment of these patients depends on an accurate diagnosis.

## Introduction

Ameloblastoma is a benign but locally aggressive odontogenic tumor of the mandible and maxilla. It represents about 1% of all jaw tumors, and 80% of ameloblastomas occur in the mandible [1]. Ameloblastomas grow slowly and, if neglected, may grow to enormous sizes, causing severe facial deformities and functional impairment [1,2]. Surgical resection with wide margins is the treatment of choice [3,4]. Radiological investigations are useful, both as aids to diagnosis and for planning surgery, an orthopantogram may reveal a "soap bubble" appearance, and an axial computed tomography (CT) scan will reveal the extent of bony and/or soft tissue involvement. Ameloblastomas may rarely degenerate into ameloblastic carcinomas.

Squamous cell carcinoma, on the other hand, is the commonest malignancy of the oral cavity, constituting about 90% of all oral cancers [5]. Most squamous cell carcinomas found in the jaws originate from lesions within the oral cavity; however, primary intra-osseous carcinoma may arise within the jaw, most likely developing from residues of odontogenic epithelium [6]. Surgical excision of resectable lesions is the mainstay of treatment. The simultaneous occurrence of squamous cell carcinoma and ameloblastoma has previously been reported [6-9]. Herein the author presents an unusual case of squamous cell carcinoma that developed in an orocutaneous fistula through a large ameloblastoma of the mandible.

## Case presentation

**Figure 1:**
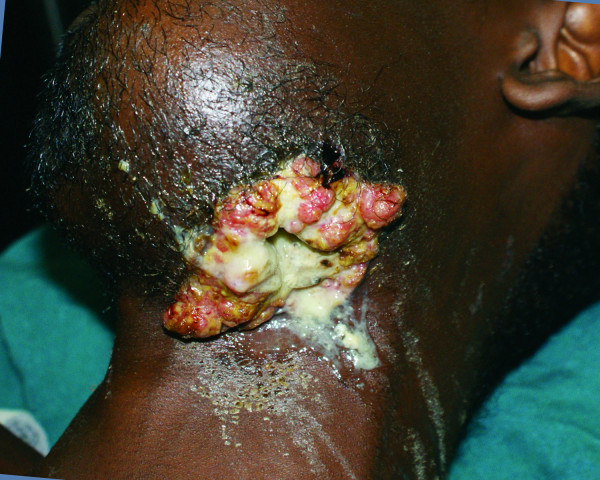
**Pre-operative image showing large left tumor with an orocutaneous fistula through which drained a copious discharge of saliva as well as liquids and food particles**.

A 35-year-old African man presented to the author's hospital with a 10-year history of a left mandibular tumor that had grown gradually over time. The tumor had ulcerated two years prior to presentation, with a resultant orocutaneous fistula through which drained saliva as well as liquids and food particles that he attempted to ingest (Figure 1), all of which produced a foul smell. Besides a history of having chewed khat for most of his adult life, the patient had no other identifiable risk factors for oral malignancy.

**Figure 2:**
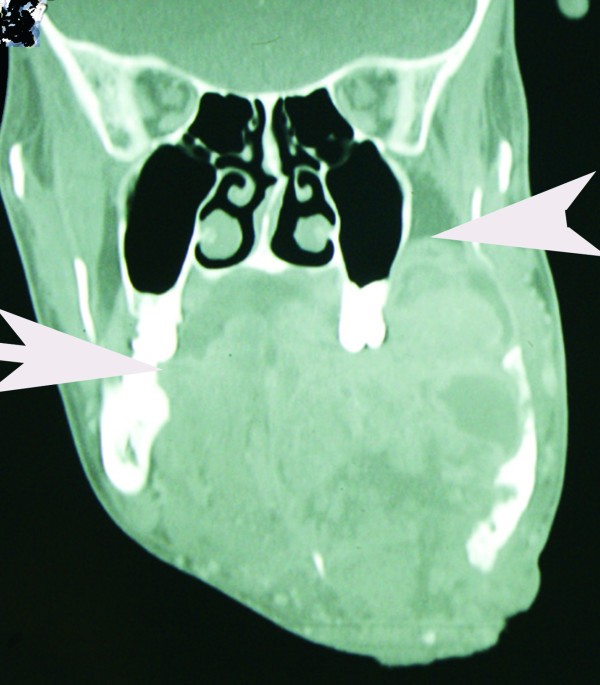
**Coronal CT scan showing extent of tumor**. Arrows indicate tumor extending into the contralateral mandible and apparently abutting the maxillary sinus. Neither the right mandible nor the maxilla was invaded by the tumor. The entire left hemimandible was involved.

His physical examination revealed a wasted appearance with a large, ulcerated left- sided mandibular tumor that emitted a purulent, foul-smelling discharge (Figure 1). A CT scan revealed a large left-sided mandibular tumor extending into the left maxilla and abutting the maxillary sinus (Figure 2), suggesting that, at most, surgical resection would be largely palliative. During surgery, a tracheostomy and a gastrostomy feeding tube were fashioned to ease post-operative airway management and nutrition delivery. The presence of an orocutaneous fistula was confirmed. The tumor was limited to the left side of the hemi-mandible with no maxillary involvement. The tumor was excised, and the resulting oropharyngeal mucosal and neck defects were reconstructed by using a left supraclavicular fasciocutaneous flap.

**Figure 3:**
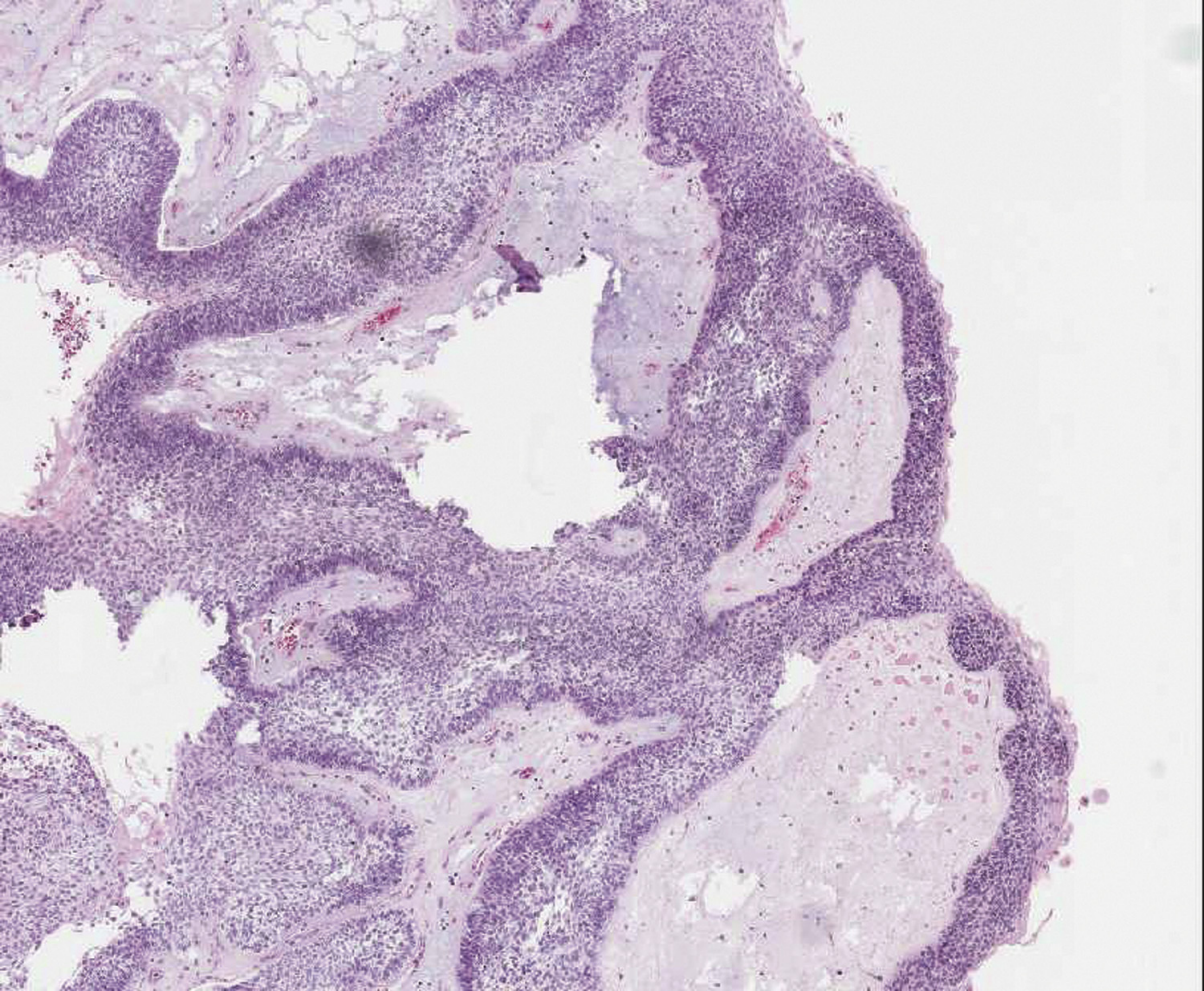
**Image showing features consistent with ameloblastoma (hematoxylin and eosin stain; original magnification, × 100 magnification)**.

**Figure 4:**
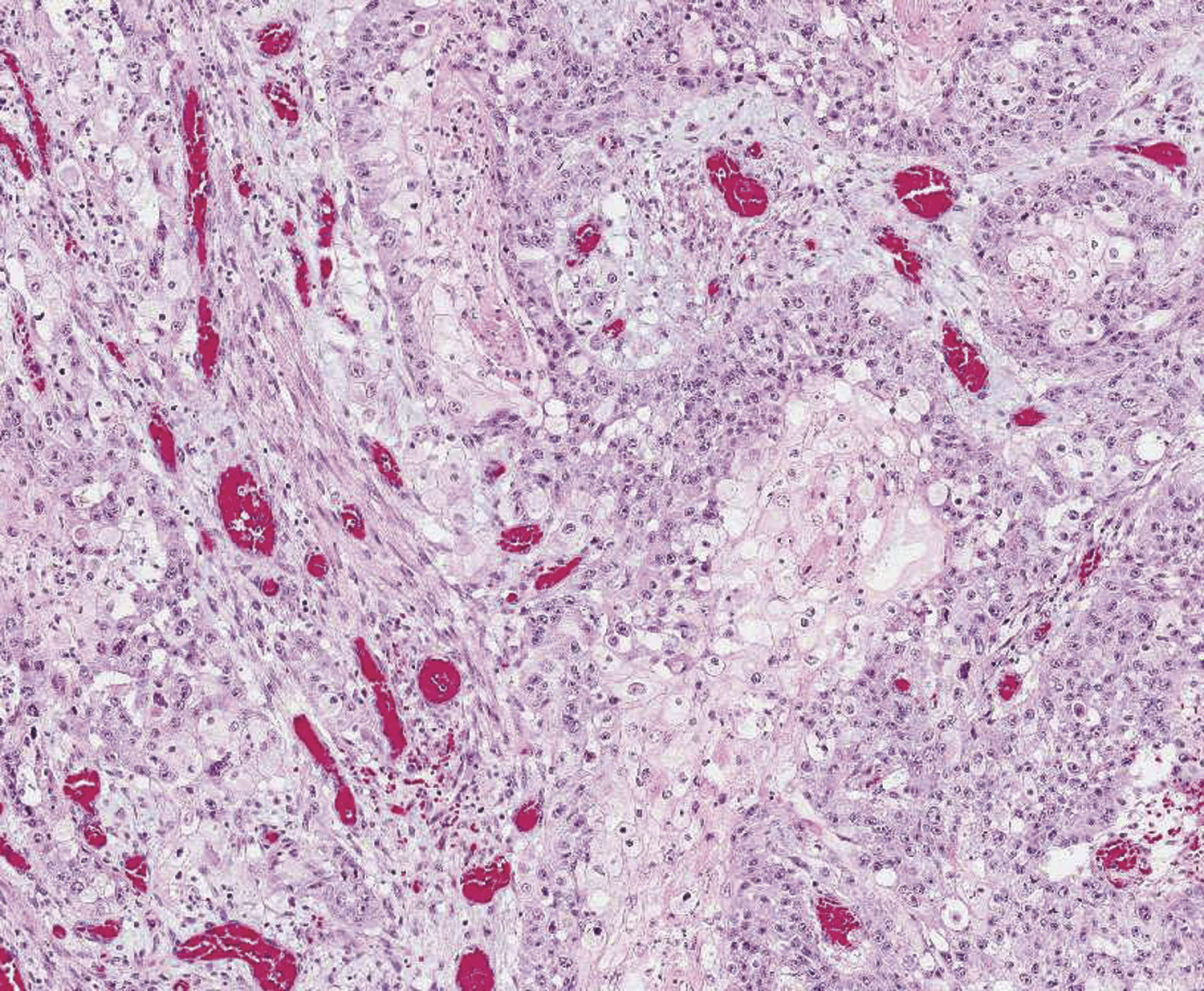
**Image showing squamous cell carcinoma in tissue from the orocutaneous fistula (hematoxylin and eosin stain; original magnification, × 200 magnification)**.

Histopathological examination of the tumor specimen revealed it to be an ameloblastoma with clear surgical margins, but it contained within it a squamous cell carcinoma limited to the orocutaneous fistula (Figures 3 and 4). There was no evidence of tumour in the submitted neck nodes.

**Figure 5:**
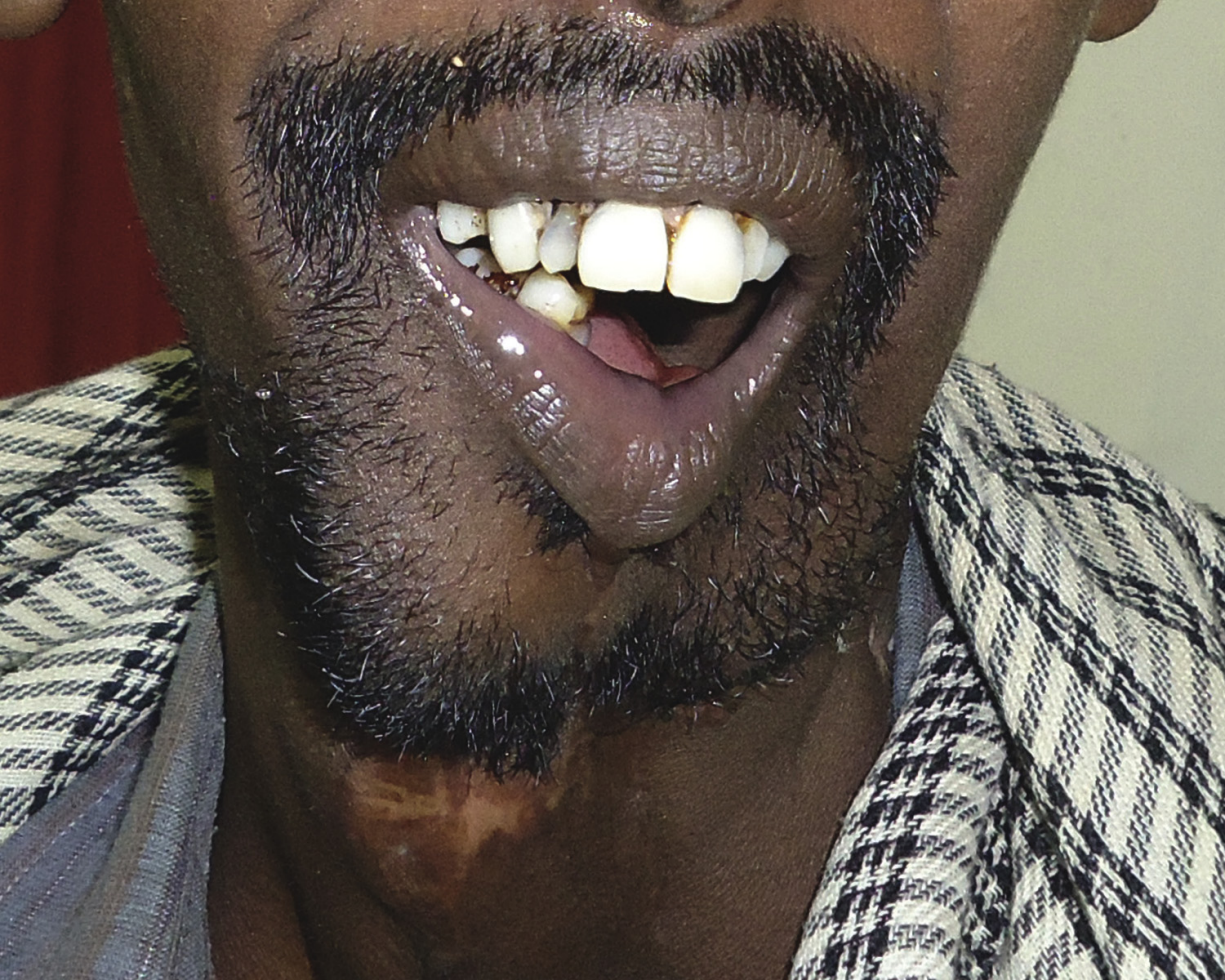
**At the patient's 13-month follow-up examination after undergoing resection, no evidence of local tumor recurrence was observed, and the patient reported excellent mastication and oral continence**.

Post-operatively, the patient did well and was offered left-sided mandibular reconstruction, but he was lost to follow-up after four months, until he re-presented to the hospital in healthy condition 13 months after surgery, with no evidence of either local recurrence or systemic tumor spread (Figure 5).

## Discussion

Tumors may grow to a size that outstrips their blood supply, leading to tumor necrosis and ulceration. If the tumor occurs in an anatomical area with two apposing epithelialized surfaces such as the oral cavity and skin, it is feasible that tumor necrosis and ulceration into both epithelia might lead to the formation of a fistula. Repeated attempts at epithelialization of the tract, with constant irritation by saliva, fluids, and oral bacteria, may lead to malignant degeneration into squamous cell carcinoma, also known as "Marjolin's ulcer." Marjolin's ulcers occur in scar tissue, classically in burn scars, but have also been described in numerous other conditions, including chronic sinuses and fistulas, such as those that occur in chronic osteomyelitis and urinary fistulas [10,11]. The fact that squamous cell carcinoma was found only along the orocutaneous fistula in this patient provides a strong basis for the hypothesis that chronic inflammation along the fistula over time led to malignant degeneration and hence to Marjolin's ulcer (Figure 1). Because of the poor prognosis associated with Marjolin's ulcers [10,11], the patient was encouraged to return for regular follow-up visits. His returns for follow-up were erratic, with no visits recorded between four months and twelve months post-operatively. The patient was noted to have gained weight, with no evidence of local or distant metastasis noted at the thirteen-month follow-up examination (Figure 5).

Hamakawa *et al*. [6] reported the case of a patient with a mandibular tumor that, upon histological examination, was revealed to be both an ameloblastoma and a squamous cell carcinoma. Tucker *et al*. [7] reported the case of a patient who had simultaneous ameloblastoma and squamous cell carcinoma in the right and left mandibles, respectively. Ueta *et al*. [8] reported the case of a patient who initially had an ameloblastoma, but after recurrence and two subsequent resections it was found to have evolved into a squamous cell carcinoma. The sources of the squamous cell carcinomas in previous reports of concurrent ameloblastoma and squamous cell carcinoma have been unclear in previous reports [7,9], while concurrent lesions in different sites [6] or tumors that were discovered subsequent to radiotherapy at the same site [9] have been described in other reports. Tucker *et al*. [7] proposed that both lesions in their patient may have arisen from one source: a radiolucent anterior mandibular lesion. Table 1 summarizes the demographics of patients found to have ameloblastoma and a concurrent or subsequent squamous cell carcinoma [6-9]. The author believes the present case report to be the first description in the English-language literature of a Marjolin's ulcer within an ameloblastoma.

**Table 1:** Demographics of patients reported with simultaneous ameloblastoma and squamous cell carcinoma of the mandible and/or maxilla

Reference	Age, years	Sex	Site	Treatment	Post-operative follow-up
Hamakawa *et al*. [6]	64	F	Left mandible	Chemotherapy followed by mandibulectomy and neck dissection	No recurrence at four years
Tucker *et al*. [7]	70	M	Right and left mandibles	Unclear	Unclear
Ueta *et al*. [8]	60	F	Right mandible	Serial excisions leading to right mandibulectomy	Lung metastasis at one year
Nishimura *et al*. [9]	52	M	Left maxilla	Radiotherapy for SCC followed by partial maxillectomy for ameloblastoma	No recurrence at 33 months
Present report	35	M	Left mandible	Mandibulectomy	No recurrence at last visit 13 months after surgery

## Conclusion

The occurrence of concurrent ameloblastoma and squamous cell carcinoma of the jaws, though previously reported, is extremely rare. Because the two lesions require different management strategies, careful histopathological examination of tumor specimens is crucial to surgical management and ultimately to clinical outcome. Marjolin's ulcers have not been previously reported to occur in tumors. This case report indicates that they can occur and that close follow-up, even in resource-poor environments, is important, because Marjolin's ulcers are generally associated with poor outcomes.

## Consent

Written informed consent was obtained from the patient for publication of this case report and any accompanying images. A copy of the written consent is available for review by the Editor-in-Chief of this journal.

## Competing interests

The authors declare that they have no competing interests.

## Authors' contributions

PMN came up with the idea for and wrote the manuscript.
